# Tin filter compared to low kV protocols - optimizing sinonasal imaging in computed tomography

**DOI:** 10.1371/journal.pone.0279907

**Published:** 2023-01-06

**Authors:** Simone Schüle, Joachim Rudolf Balthasar Strobel, Kai Johannes Lorenz, Meinrad Beer, Carsten Hackenbroch

**Affiliations:** 1 Department of Diagnostic and Interventional Radiology and Neuroradiology, German Armed Forces Hospital Ulm, Ulm, Baden-Wurttemberg, Germany; 2 Department of Otorhinolaryngology and Head and Neck Surgery, German Armed Forces Central Hospital Koblenz, Koblenz, Rhineland-Palatinate, Germany; 3 Department of Radiology, University Hospital of Ulm, Ulm, Baden-Wurttemberg, Germany; Affiliated Hospital of Nanjing University of Chinese Medicine: Jiangsu Province Academy of Traditional Chinese Medicine, CHINA

## Abstract

**Objectives:**

Paranasal sinus imaging due to chronic inflammatory disease is one of the most common examinations in head and neck radiology with CT imaging considered the current gold standard. In this phantom study we analyzed different low dose CT protocols in terms of image quality, radiation exposure and subjective evaluation in order to establish an optimized scanning protocol.

**Methods:**

In a phantom study, an Alderson phantom was scanned using 12 protocols between 70–120 kV and 25–200 mAs with and without tin filtration. For all datasets, iterative reconstruction was used. Data were objectively evaluated (image noise, (dose-weighted) contrast-to-noise ratio) and for subjective evaluation an online survey using a Likert scale was performed to reach a large group of clinically experienced reader (n = 62). The protocol was considered diagnostically insufficient if the median score was 4 and above and if more than 10% of raters scored 4 and above on the Likert scale. For an interreader agreement an ICC was calculated. To compare clinical value in relation to the applied dose and the objective image parameters, we calculated a figure of merit (FOM) and ranked the protocols accordingly.

**Results:**

There was an overall moderate agreement between the 62 readers for the 12 examined CT protocols. In this phantom study, protocols with 100 kV with spectral shaping and 50–100 mAs obtained the best results for its combination of dose, image quality and clinical information value for diagnosing sinusitis (FOM 1^st^– 2^nd^ place) with the 70 kV and 50 mAs as a good alternative as well (Sinusitis: FOM shared 2^nd^). For preoperative planning, where a higher dose is necessary, 100 kV with spectral shaping and 100 mAs achieved the overall best results (FOM 1^st^ place) with 70 kV and 50 mAs ranking 4^th^.

**Conclusion:**

100-kV protocols with spectral shaping or low kV protocols (70 kV) with a similarly low dose showed the best figure of merit for imaging sinonasal disease and preoperative planning. With modern scanner technology available, spectral shaping or low KV protocols should be used for sinusitis imaging.

## Introduction

(Chronic) rhinosinusitis is a very common disease in population [1, 2]. While an uncomplicated sinusitis does not require radiologic imaging, it is indicated when symptoms are recurrent or refractory despite adequate treatment [3]. CT imaging should also be obtained early in immunocompromised patients with an acute onset or worsening chronic clinic [4]. The most important imaging modality hereby is the low dose CT examination due to its three-dimensional imaging, good resolution of even small bony structures and cost efficiency [5, 6]. In recent years, advancements of CT scanners detectors [7] and the reduction of the tube voltage from 120 kV and 100 kV down to 70 kV has resulted in dose-reduced protocols for sinonasal imaging [8–13]. Lowering of the tube current significantly reduces the exposure with the backside of increased image noise levels. Because examinations of the paranasal sinus are high-contrast examinations and the clinical objectives are limited, higher image noise with reduced image quality is acceptable [1, 6, 14].

The use of modern iterative reconstruction methods (IR) to replace standard filtered back projection (FBP) has also significantly reduced the dose exposure [10, 15–18]. Another new approach is the use of filters in computed tomography (spectral shaping) [17, 19, 20]. Hereby, a tin layer is added right beneath the x-ray tube, which removes softer fractions of the x-ray spectrum. The softer fractions of the x-ray spectrum contribute little to image quality but increase the radiation dose, especially in superficial tissues [21]. This is of particular importance, because sinonasal diseases may require repetitive imaging in young, otherwise healthy patients with radiosensitive, superficial organs within the field of direct exposure (e.g. eye lenses) [22]. Also, through tin filtration the mean photon energy of the x-ray spectrum increases (100 kV = 58.7 keV, 120 kV = 64.2 keV, Sn 100 = 76 keV) [23], resulting in fewer beam hardening of the x-ray spectrum when passing through a patient’s body. Consequently, equal beam hardening effects can be observed with a reduced dose of the Sn 100 kV protocol as with the 120 kV protocol [24].

In this experimental phantom study, various established protocols as well as new ultra low dose protocols were tested and compared with special focus on spectral shaping. By combining objective image parameters with the subjective evaluation by a cohort of radiologists and referring physicians, optimal scanner parameters for diagnostic imaging of the paranasal sinus regarding CTDI_Vol_, tube voltage (kV) and tube current (mAs) were determined.

## Material and methods

### Image acquisition

Ethical approval for this phantom study was waived by institutional review board. An Alderson phantom was scanned on a 3rd generation DSCT scanner (CT Somatom Force; Siemens Healthineers) using various CT- protocols, which were recommended by literature, were newly established or were the in-house standard protocol (Table 1).

**Table 1:** CT acquisition parameters.

Tube voltage (kV)		Tube current (mAs)	Tin filter	CTDI_Vol_ (mGy)	DLP (mGy*cm)	Effective dose (mSv)	Collimation and Slices (mm x #)	Pitch	Rotation time (1/s)	Scan duration (sec)	Protocol origin
	70	50	-	1.40	21.0	0.040	0.6 x 96	0.8	1	2.4	[6]
	70	75	-	2.11	30.9	0.059	0.6 x 96	0.8	1	2.4	[8]
	100	25	-	2.11	30.6	0.058	0.6 x 96	0.8	1	2.4	in-house/[6]
	100	100	-	8.78	128.0	0.243	0.6 x 96	0.8	1	2.4	in-house/[6]
	120	20	-	2.71	39.6	0.075	0.6 x 96	1.0	1	1.9	in-house
	120	40	-	5.50	81.0	0.154	0.6 x 96	1.0	1	1.9	[8]
	120	70	-	9.82	145.2	0.279	0.6 x 96	1.0	1	1.9	vendor
	100	25	X	0.20	2.8	0.005	0.6 x 96	0.6	0.5	1.6	[6]
	100	50	X	0.43	5.5	0.010	0.6 x 96	0.6	0.5	1.6	[6]
	100	100	X	0.85	11.3	0.021	0.6 x 96	0.6	0.5	1.6	in-house/[6]
	100	150	X	1.30	16.8	0.032	0.6 x 96	0.6	0.5	1.6	in-house/[6]
	100	200	X	1.73	22.5	0.043	0.6 x 96	0.6	0.5	1.6	[6]

Protocols are color coded based on their tube voltage and tin filtration. Red = 70 kV, green 100 kV, yellow = 120 kV and blue = Sn 100 kV. The numbers in the column “Protocol origin” refer to the corresponding references in the reference list. CTDI_Vol_ = computed tomography dose index, DLP = dose length product, # = number.

To compare CDTI_Vol_ given by the scanner for the different scans, the tin filter protocols had to be adjusted by the factor of 2.5 according to the manufacturer manual and Lell et al‥. While all other scans were calibrated on a 16-cm phantom, the tin filter protocols at this time were calibrated on a 32-cm body phantom, because those protocols originated from thoracic imaging. Meanwhile, the manufacture now provides tin filter protocols calibrated to a 16-cm phantom. For better comparison with literature the effective dose was calculated using the following formula [25]:

Effectivedose=doselengthproduct×conversionfactork

where conversion factor k = 0.0019 [26, 27].

### Image postprocessing

The acquired data were reconstructed in all three spatial planes (axial, coronal, sagittal) in bone window with a slice thickness of 1 mm (kernel: Hr64, increment: 0.75mm, matrix size: 512 x 512 voxels) as well as axial in soft tissue window with a slice thickness of 4 mm (kernel: Hr36). Reconstruction was performed with an iterative reconstruction procedure of the 3rd generation: ADMIRE (Advanced Modeled Iterative Reconstruction, Siemens Healthineers), level 3 for bone and for soft tissue reconstructions. The window level of the images was C 1200/W 4000 for bone window and C 70/W 370 for soft tissue window.

### Objective evaluation

All protocols were evaluated based on triplicate region of interest (ROI) measurements in axial plane (S1 Fig) The mean HU-value and the standard deviation (Std.Dev.) of cortical bone, hard rubber coating of the phantom (which simulates soft tissue) and the ambient air were determined. The ROIs were placed by an experienced radiology resident using the software SyngoVia (Siemens Healthineers). A copy-paste function and automatic image correlation ensured that the individual ROIs were placed at the identical areas.

Based on these results, the contrast-to-noise ratio (CNR) was calculated using the following formula [28, 29]:

$$CNR=\frac{Mean{\left(HU\right)}_{Bone}-Mean{\left(HU\right)}_{Hard\;Rubber}}{\text{Standard}\;\text{deviation}\;(\text{Mean}\;{\text{HU})}_{Air}}$$

In order to correct the radiation dose difference between the scans (esp. high dose vs low dose) dose-weighted contrast-to-noise ratio (CNRD) were calculated, using the formula [30]:

$$CNRD=\frac{CNR}{\sqrt{CTDI_{Vol}}}$$

### Online survey (subjective image evaluation)

**Fig 1:**
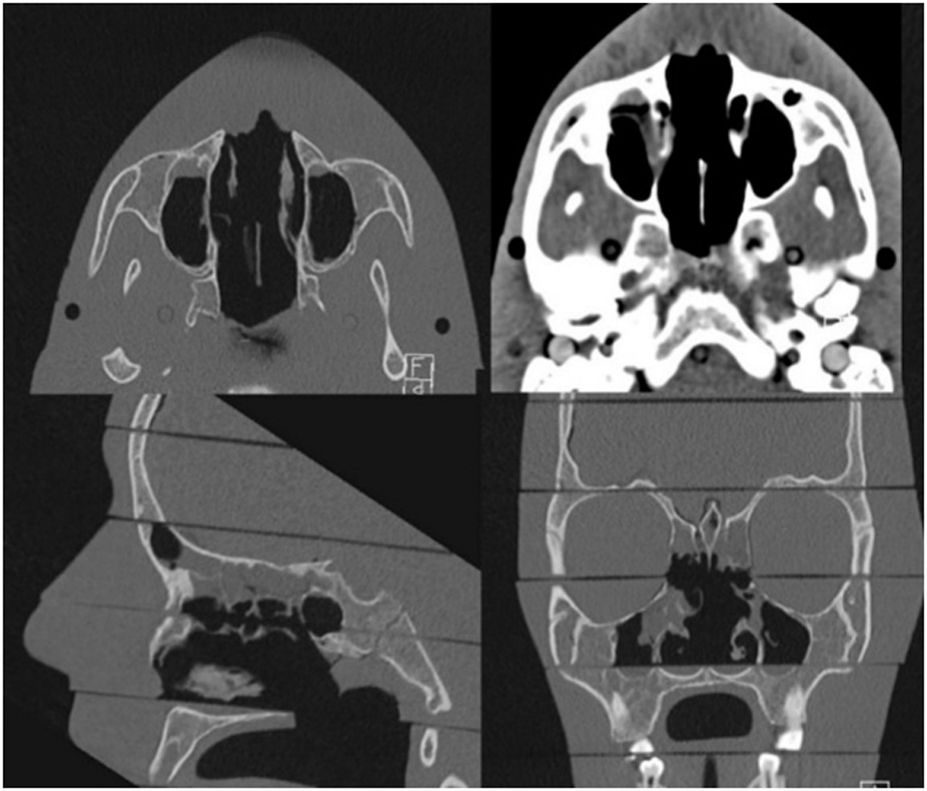
Example image panel of the online survey consisting of three CT-images in bone window in all three spatial directions and a single axial slice in a soft tissue window.

To reach a large group of experienced readers in sinonasal disease, an online survey was performed. Herefor, Ct-images were imported into the Osirix software (Pixmeo, Bernex Switzerland) and converted into lossless JPEG format. 3 CT-images in all 3 spatial directions (bone window) and a single axial slice in a soft tissue window were used per scan (Fig 1).

In this online survey (Electric Paper Evaluationssysteme GmbH, Lüneburg, Germany) a total of 62 participants took part. Of these, 60 participants (97%) were radiologists/neuroradiologists and two (3%) otorhinolaryngologists, with 22 (34%), 32 (52%) and 9 (14%) of the physicians being residents, fellows and attendings, respectively.

Participants were asked several questions. The questions concerning sinonasal inflammatory disease imaging were:

- Is image quality sufficient for the diagnosis of sinusitis?
- Is surgery planning possible based on the quality of the images?

CT-image evaluations were subjectively conducted using a 5-item Likert scale (1 = best, 5 = worst). Readers were blinded to protocol information. We defined any median score on the Likert scale of 4 and above as diagnostically insufficient. Median scores of 3 were still satisfactory, however with impaired quality (S1 Table). If more than 10% of raters scored 4 and above on the Likert scale, then the protocol was considered clinically inadequate and was excluded from further analysis.

### Figure of merit

To be able to further analyze and better compare clinical value in relation to the applied dose, we calculated a figure of merit (FOM) according to the following formula [31, 32]:

$$FOM=\frac{{\left(mean\;value\;Likert\;scale\right)}^{2}\times standard\;deviation\;of\;air\times CTDI_{Vol}}{10}$$

### Statistics

Descriptive statistics were performed using Excel (Microsoft, Redmond, United States). Data were reported as mean ± standard deviation or, where applicable, as median + interquartile range. The graphical representations were performed using PowerPoint (Microsoft, Redmond, United States) and R (Version 4.2.1, Packages: plot3D, ggplot2).

For an interreader agreement, a two-way mixed, agreement, single-measures intra-class correlation (ICC, R package: psych) was calculated and presented with 95% confidence intervals (CI). Results were assigned to poor (< 0.5), moderate (0.50–0.75), good (0.75–0.9) and excellent (> 0.90) interreader agreement [33]. Further statistical tests were not performed because only one phantom was scanned and therefore statistical tests should be omitted.

## Results

### Objective evaluation

**Fig 2:**
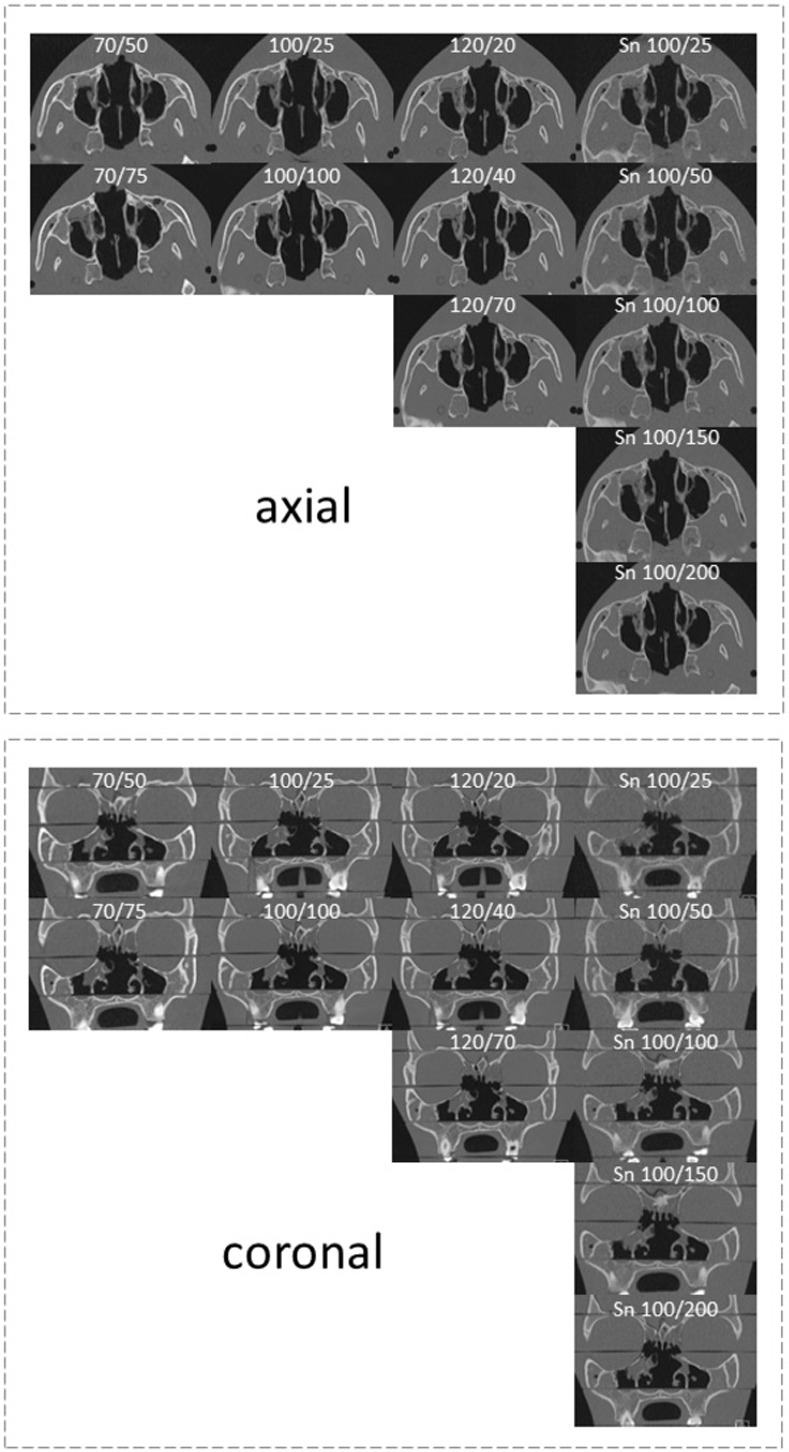
Example images of all examined protocols of the phantom study. Shown are CT-images in axial (upper panel) and coronal (lower panel) orientation. The used voltage (kV) and current (mAs) of each protocol are given at the top. Sn = tin filtration.

**Fig 3:**
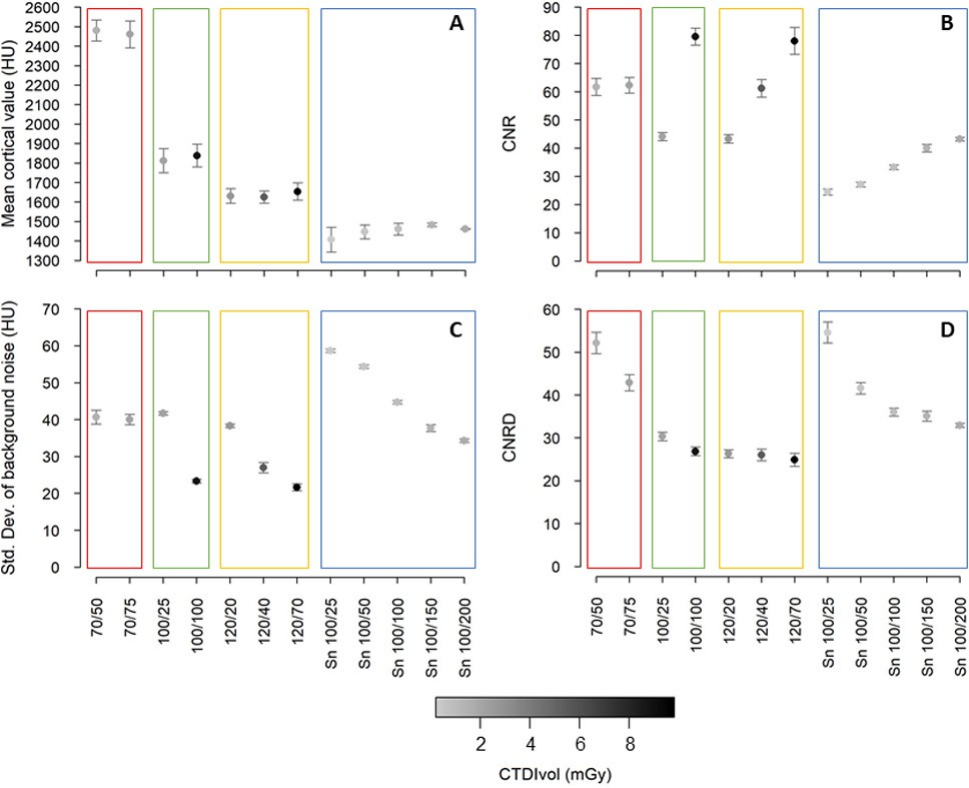
Scatterplot of objective image evaluation. Shown are mean cortical values (A), CNR (B), standard deviation of background noise (C) and CNRD (D). Symbols represent the mean and error bars the standard deviation of triplicate ROI measurements. The symbol of each protocol is color coded according to the color scale based on the CTDIVol on the bottom of the figure: the darker the grey, the higher the CTDIVol. Colored rectangles refer to the different tube voltages examined: Red = 70 kV, green 100 kV, yellow = 120 kV and blue = Sn 100 kV. CNR = contrast-to-noise ratio, CNRD = dose-weighted contrast-to-noise ratio, CTDIVol = computed tomography dose index, HU = hounsfield units, ROI = region of interest, Std.Dev. = standard deviation, Sn = tin filter.

The HU of cortical bone determined in a defined region of interest (ROI) was highest in the low kV examinations (Figs 2 and 3A) with70 kV (2471 ± 10 HU). It decreased in higher kV scans, with 100 kV showing higher HU values (1825 ± 13 HU) than 120 kV (1636 ± 12 HU). Spectral shaping protocols scored lowest (1452 ± 25 HU).

Tin protocols achieved low image noise at low dose (CTDI_Vol_ 0.2–1.73 mGy), but exhibited low CNR due to low cortical density compared to all other protocols (Fig 3B and 3C and S2 Table). The low-kV examinations (70 kV) were associated with a low dose (CTDI_Vol_ 1.4–2.11 mGy) compared to 100/100 protocol and 120 kV protocols with very high CNR and very low image noise. The 100/100 and 120/70 protocols showed the highest CNR and lowest image noise, but also lead to the highest dose exposure (CTDI_Vol_ 8.78–9.82 mGy).

The 70 kV protocols and the tin filter protocols with 25 and 50 mAs showed the highest dose-weighted contrast-to-noise ratios (CNRD) either due to their high contrast or low exposure (Fig 3D). The other tin filter protocols followed in descending order of increasing tube current, succeeded by the 100 kV protocols without tin filters. The 120 kV protocols showed the lowest CNRD values.

### Subjective evaluation

#### Interreader agreement

There was an overall moderate agreement between the 62 readers for the clinical question sinusitis diagnostic (κ = 0.59 (CI 0.44; 0.78)) and preoperative evaluation (κ = 0.58 (CI 0.42, 0.77)) of the 12 examined CT protocols.

#### Sinusitis diagnostic

**Fig 4:**
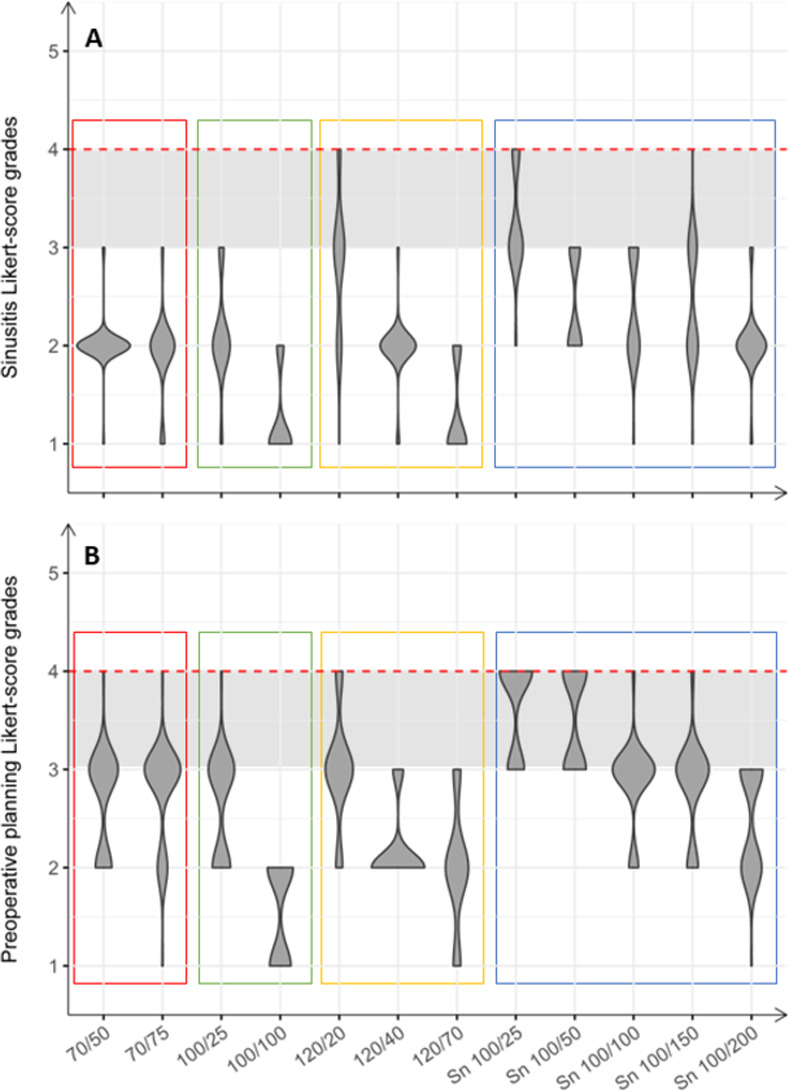
Violin pot of subjective image evaluation of sinusitis diagnosis (A) and preoperative planning (B). The horizontal red dotted line showed the Likert grade above which the diagnostic image quality is insufficient for clinical use. Grey horizontal box shows area in which image quality is impaired. Colored rectangles refer to the different tube voltages examined: Red = 70 kV, green 100 kV, yellow = 120 kV and blue = Sn 100 kV.

More than 10% of the raters evaluated the Sn 100/25 and the 120/20 protocol (median 3, IQR = 1) as a 4 on the Likert scale. Hence, the Sn 100/25 and the 120/20 protocols were not sufficient for diagnosing sinusitis and thus excluded from further analysis. All remaining protocols were rated as sufficient for diagnosing sinusitis (Fig 4A and S3 Table). The 120/70 and 100/100 protocol were rated best, but consequently had the highest dose with a CTDI_Vol_ of 9.8 mGy and 8.8 mGy, respectively. The remaining protocols were scored equally (median 2, IQR 0/1), with protocol 120/40 applying the highest (CTDIVol: 5.5 mGy) and protocol Sn 100/50 applying the lowest (CTDIVol: 0.2 mGy) dose.

#### Preoperative planning

Scores for preoperative planning increased on the Likert scale compared with scores for sinusitis (Fig 4B). Ultra-low dose tin filter protocols (Sn 100/25, Sn 100/50) with CTDI_Vol_ of 0.2 mGy and 0.4 mGy were rated worst (median: 4, IQR 1), therefore found insufficient for clinical usage and thus excluded from further analysis. Additionally, more than 10% of the raters evaluated the 120/20 protocol (median 3, IQR 0) as a 4 on the Likert scale. Hence, the 120/20 protocol was also excluded from further analysis. Again, the 120/70, 120/40 and 100/100 protocol (median 2, IQR 0/1) were rated best, due to their high dose. All remaining protocols (median 3, IQR 0/1) were rated just sufficient for preoperative planning with the Sn 100/200 protocol showing the best results among the low-dose protocols below 2.7 mGy.

#### FOM

Protocols using spectral shaping showed overall the lowest FOMs (Table 2). The Sn 100 kV / 50 mAs protocol and the Sn 100 kV / 100 mAs had the lowest FOM among protocols with sufficient image quality for sinusitis diagnostic or preoperative planning, respectively. The FOM of 70-kV protocols were low for the diagnosis of sinusitis, similar to those of protocols with tin filters, but for preoperative planning, the FOM were higher than for protocols with tin filters. The high-dose protocols had the highest FOMS due to their high dose.

**Table 2:** FOM results for evaluation of sinusitis and preoperative planning.

Protocol		FOM					
		Sinusitis			Preoperative imaging		
		Mean	±	Std. Dev.	Mean	±	Std. Dev.
	Sn 100/25	13	±	4	16	±	4
	**Sn 100/50**	**14**	**±**	**6**	29	±	8
	**Sn 100/100**	**23**	**±**	**10**	**32**	**±**	**9**
	**Sn 100/200**	**25**	**±**	**9**	**39**	**±**	**15**
	**Sn 100/150**	**30**	**±**	**14**	**40**	**±**	**14**
	**70/50**	**23**	**±**	**6**	**42**	**±**	**15**
	**100/100**	**34**	**±**	**26**	**54**	**±**	**31**
	**100/25**	**42**	**±**	**20**	**62**	**±**	**24**
	**70/75**	**33**	**±**	**13**	**67**	**±**	**21**
	**120/40**	**59**	**±**	**17**	**72**	**±**	**27**
	120/20	40	±	29	96	±	55
	**120/70**	**89**	**±**	**39**	**97**	**±**	**36**

The lower the value, the better the result. Only values in bold fulfill the required image quality for diagnosing sinusitis and preoperative planning. Protocols are ranked from top to bottom according to the preoperative planning FOM results from best to worst. Protocols are color coded based on their tube voltage and tin filtration. Red = 70 kV, green 100 kV, yellow = 120 kV and blue = Sn 100 kV. FOM = figure of merit, Std. Dev. = standard deviation.

## Discussion

The low-dose CT and ultra-low dose CT is currently considered the gold standard for visualizing the paranasal sinuses [5]. Modern detectors with increasing photon yields, iterative reconstruction techniques, lower tube voltage and tin filtration have enabled even greater dose savings in recent years [7–13, 15–20]. Also, applying dose reduction to the topogram (e.g., through tin filtering) keeps the combined dose to a minimum, especially when performing ultra-low dose scans. In our study, we compared different scanning protocols for imaging the paranasal sinuses with and without tin filtration, most of which had a CTDI_Vol_ between 0.2 mGy and 2.7 mGy. A group of 62 otolaryngologists and (neuro)radiologists rated CT images for image quality for the diagnosis of inflammatory sinus disease and use for preoperative planning on a 5-point Likert scale ((1 = best, 5 = worst). Our key findings can be summarized as follows. In this phantom study, the Sn 100 kV/100 mAs protocol (CTDI_Vol_ 0.85 mGy, ED 0.02 mSv) obtained the best results for sinusitis diagnostic and preoperative planning imaging in terms of objective parameters, dose exposure, and clinical consideration. For the diagnosis of inflammatory sinus disease alone, the Sn 100kV/50 mAs protocol (CTDI_Vol_ 0.43 mGy, ED 0.010 mSv) is sufficient. 100 kV and 120 kV protocols scored worst. Excessive dose reduction ≤ 0.8 mGy for preoperative planning resulted in insufficient image quality with consecutive re-imaging causing additional dose exposure to the patient. The Sn 100/25 protocol was evaluated as non diagnostic for sinusitis, precluding further dose reduction. Lower current levels may be sufficient for follow-up studies. However, this statement is beyond the results of our study and needs to be investigated further.

Our findings are in accordance with other studies addressing imaging of the paranasal sinuses with tin filtration. In retrospective patient studies with ≥ 100 patients, it has been shown that a) the Sn 100 kV/35 mAs protocol is sufficient for the diagnosis of inflammatory diseases of the paranasal sinuses [26], b) the Sn 100 kV/150 mAs protocol results in sufficient image quality for imaging of the paranasal sinuses at lower doses compared with a 100 kV/50 mAs protocol [20], and c) the Sn 100 kV/200 mAs protocol results in comparable image at a 17% lower dose than the 100 kV/25 mAs protocol [19]. However, in patient studies, a systematic investigation of the minimum required dose exposure for diagnostic image quality is difficult because due to ethical standards often two patient groups representing two protocols are compared [19, 20] or even no control group is available [26]. Phantom and cadaver studies allow a more systematic evaluation of optimal scanner parameter settings for specific clinical questions, e.g. inflammatory diseases of the paranasal sinuses or preoperative planning, without exposing the patient to an unnecessary radiation dose. As such, the findings of Lell et al. in their cadaver study are similar to ours [6]. They concluded, that for preoperative planning the Sn 100 kV / 150 mAs protocol and for diagnosing sinusitis the Sn 100/ 25 mAs is sufficient [6]. In comparison to Lell et al. we additionally evaluated objective image parameters and also correlated them to subjective image assessment and applied dose (FOM). Moreover, the quantity of raters (n = 62) was much greater compared to Lell et al. (n = 2). While most of our results are comparable to those of Lell et al, the 70-kV protocols in particular benefited from these additional assessments regarding the diagnosis of sinusitis. Other studies also describe the diagnostic usefulness of 70-kV protocols for the diagnosis of sinusitis [8]. If 70-kV scans on the scanner are not possible, even 80-kV protocols allow low-dose imaging of the paranasal sinuses [12]. Our study has thus contributed considerably to the advancement of low-dose imaging of the paranasal sinuses.

MRI can also be used for diagnosing sinusitis, in particular with high-resolution T2w sequences. However, the method is not suitable for imaging fine bony structures, which is particularly important for surgical planning. However, MRI is the gold standard in tumor diagnostics of the midface thanks to high-contrast imaging of soft tissue. With excellent detail of bony anatomy, cone beam CT (CBCT) additionally plays an important role in imaging of the paranasal sinuses for out clinic patients but is of very limited soft-tissue information. In CBCT, dose exposure is considered low [34–37]. However, this is only true when compared to CT examinations with dose exposures as low as 0.27 mSv [35]/ 0.6 mSv [34] and 4 mGy [37] and not when compared to sophisticated ultra-low-dose CT protocols [6]. Conventional radiography no longer plays any real role in diagnosing the paranasal sinuses as the resulting images lack the detail and image quality required to visualize the relevant complex anatomy. Low-dose high-resolution computed tomography (CT) has been established as the best method currently available for imaging the sinus system [5, 38, 39].

The use of spectral shaping leads to a homogenized, hardened radiation which has an intrinsically lower image noise than non-tin filter examinations, which benefits the image quality. This has been confirmed in several studies [19, 32, 40, 41]. At the same time, however, the absence of the softer radiation components leads to a loss of contrast, with a corresponding loss of CNR [20, 32, 40, 41]. This decrease in contrast is usually not important in high-contrast examinations such as imaging bone or lung structures. However, from our own experience, tin filter examinations of the paranasal sinuses with considerable mucosal swelling can lead to a poorer delineation of the finest bony structures, which can only be compensated by increasing the dose. This disadvantage of tin filtered protocols is aggravated when using Sn 150 kV protocols rather than Sn 100 kV protocols. Furthermore, it must be mentioned that the radiologist reporting the findings first has to get used to the low-contrast image impression. Low kV protocols (e.g. 70kV), on the other hand, intrinsically exhibit a very high soft tissue contrast and also present the finest bony structures with a high contrast, which was also confirmed by Bodelle et al. [8]. Surprisingly, a presumed increased image noise compared to the higher kV values could not be proven in our study. Bodelle describes an increased image noise when using 70 kV protocols. In his study, however, the image noise of all protocols was much higher than in our study, which can be explained by the more modern scanner technology, the more sensitive detector and the different reconstruction parameters (layer thickness, kernel, iterative reconstruction level) we used. In our study the 70 kV low dose protocols show a CNR that is nearly as high as in the high dose protocols at 100 kV and 120 kV. In contrast, the tin filter studies perform poorly in the calculation of the CNR due to their low contrast. Nevertheless, in our opinion, image noise is a more reliable parameter for assessing image quality when comparing tin with non-tin filter scans, especially for high contrast examinations.

While high image noise and reduced image quality may be acceptable in the pre-surgical assessment of uncomplicated sinonasal inflammatory disease, it cannot be tolerated in surgical planning, where the cribriform plate, lamina papyracea, orbital walls and neurovascular channels must be clearly identifiable [6, 42]. In addition, it must still be possible to assess the soft tissue in order to be able to delineate secondary findings.

Therefore, the dose cannot be reduced beyond a certain limit. In our study, the image quality of the Sn 100 kV / 25 mAs and Sn 100 kV / 50 mAs was non-sufficient for the diagnosis of sinusitis and preoperative planning together. Our study showed that a dose with a tube-current time product of at least 50 mAs is required for satisfactory diagnosis of sinusitis alone. However, since a positive diagnosis often leads to a surgical consequence, the initial dose should be sufficient to allow preoperative planning (computer-assisted if necessary) to avoid unnecessary repeat scans. In combination with our online survey, a value of around 100mAs with tin filtration at 100 kV is recommended for a satisfactory evaluation of all clinically relevant issues concerning sinusitis diagnostics and preoperative planning.

### Limitations

The main limitation is the small number of phantoms studied, which we tried to compensate by using a large number of readers to obtain more reliable and reproducible results. Additionally, the data obtained in our study only applies to the type of high-end scanner we used and cannot be easily transferred to other scanners. Up to now, there are only few spectral shaping scanners available, but the number is rising. The same is true for 70 kV protocols for most scanners. Nevertheless, 80kV protocols can be achieved easily. Ultimately, the aim is to monitor the dose on your own scanner constantly and adjustment and reduce it with all the available options. With the new photon counting detector technique now available even greater dose savings can be achieved

## Conclusion

Our phantom study showed that 100 kV tin-filter imaging and low kV scans wit 70 kV performed best in terms of dose reduction while keeping image quality high. The dose reduction achieved with tin filtration was especially great. With modern scanner technology available ultra-low dose scans with spectral shaping or low KV protocols should be used for sinusitis imaging. However, it is important to scan with a sufficiently high dose to avoid CT images with unsatisfactory image quality With the novel photon counting technique, even greater dose savings are possible in CT imaging of the paranasal sinuses [43].

## Supporting information

S1 Table Likert score grades definition for sinusitis diagnostics and preoperative planning. (XLSX)

S2 Table Raw data of objective image evaluation per protocol based on triplicate ROI measurements. Protocols are color coded based on their tube voltage and tin filtration. Red = 70 kV, green 100 kV, yellow = 120 kV and blue = Sn 100 kV. CNR = contrast-to-noise ratio, CNRD = dose-weighted contrast-to-noise ratio, HU = hounsfield units, ROI = region of interes, Std.Dev. = standard deviation, Sn = tin filter.
(XLSX)

S3 Table Number of raters per protocol and Likert score in subjective image assessment for sinusitis diagnosis and preoperative planning. Protocols are color coded based on their tube voltage and tin filtration. Red = 70 kV, green 100 kV, yellow = 120 kV and blue = Sn 100 kV.
(XLSX)

S1 Fig Example of ROI measurements in a CT image of the Alderson phantom for objective image evaluation. The yellow, purple and red circles represent representative ROI measurement positions for air, soft tissue and bone structures, respectively.
(TIF)
