# Supplementary figures and images for: Tin filter compared to low kV protocols - optimizing sinonasal imaging in computed tomography
Source: PLoS One. 2023 Jan 6;18(1):e0279907. doi: 10.1371/journal.pone.0279907 (PMC9821404; doi:10.1371/journal.pone.0279907)

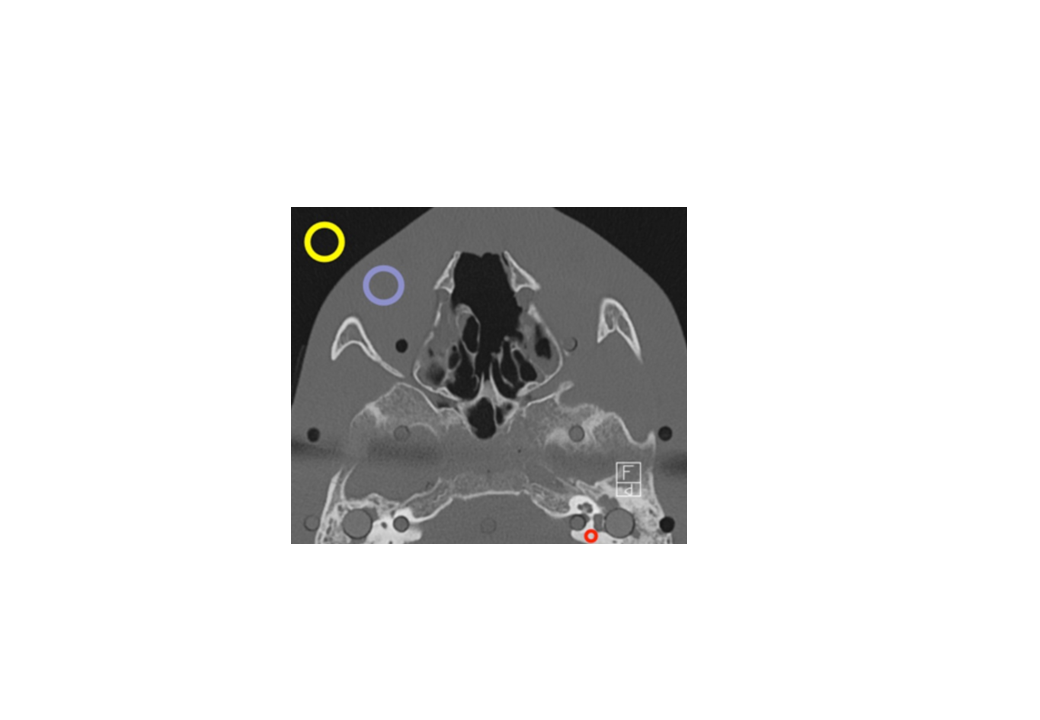

Supplement: S1 Fig — The yellow, purple and red circles represent representative ROI measurement positions for air, soft tissue and bone structures, respectively. (TIF) [file pone.0279907.s004.tif]
